# Hepatic Steatosis, Carbohydrate Intake, and Food Quotient in Patients with NAFLD

**DOI:** 10.1155/2013/428542

**Published:** 2013-05-02

**Authors:** Concepcion Gonzalez, Victor de Ledinghen, Julien Vergniol, Juliette Foucher, Brigitte Le Bail, Sabrina Carlier, Elisa Maury, Henri Gin, Vincent Rigalleau

**Affiliations:** ^1^Pôle Hépatogastroentérologie, Endocrinologie et Nutrition, CHU de Bordeaux, Avenue de Magellan, 33600 Pessac, France; ^2^Service de Pathologie, CHU de Bordeaux, France; ^3^INSERM, ISPED, Centre INSERM U897-Epidemiologie-Biostatistique, 33000 Bordeaux, France

## Abstract

Is steatosis related to the spontaneous carbohydrate intake in patients with NAFLD? We performed dietary records for 24 patients with NAFLD, 3 months after their liver biopsy was performed and before the deliverance of a dietary advice. The food quotient, indicator of the proportion of calories from carbohydrates, was calculated as (1.00×%  calories from carbohydrates/100) + (0.70×%  calories from lipids/100) + (0.81×%  calories from proteins/100). The associations between diet variables and steatosis% on the hepatic biopsies were tested by regression analysis, and diet variables were compared according to the presence of fibrosis. The subjects displayed a large range of steatosis, 50.5% ± 25.5 [10–90], correlated with their energy intake (1993 ± 597 kcal/d, *r* = 0.41, *P* < 0.05) and food quotient (0.85 ± 0.02, *r* = 0.42, *P* < 0.05), which remained significant with both variables by a multivariate regression analysis (*r* = 0.51, *P* < 0.05). For the 17/24 patients with a hepatic fibrosis, the energy intake was lower (fibrosis: 1863 ± 503 versus others: 2382 ± 733 kcal/d, *P* < 0.05), and their food quotients did not differ from patients without fibrosis. Hepatic steatosis was related to the energy and carbohydrate intakes in our patients; the role of dietary carbohydrates was detectable in the range of usual carbohydrate intake: 32% to 58% calories.

## 1. Introduction

The high prevalence of nonalcoholic fatty liver diseases (NAFLD) is now well recognized, involving 15% (ultrasound study in China [1]) to 34% (MR spectroscopy in the USA [2]) of adults. Not only serious late consequences, as liver fibrosis [3] and hepatocellular carcinoma [4], but also high rates of cardiovascular events [5], make NAFLD a relevant public health issue. Lifestyle modifications, predominantly dietary, are considered as the first line of the therapy [6]; however, beside the importance of losing excessive weight, the dietary counselling for NAFLD is not consensual, as reflected by several recent reviews [7–9]. The reduction of liver fat is a logical objective, 26% of which arise from De Novo Lipogenesis and 15% from the diet, so less dietary carbohydrates and/or lipids may help [10]; however, their proportion is debatable.

The Nutrition and Hepatology Teams of the Centre Hospitalier Universitaire de Bordeaux cooperatively follow patients with NAFLD: once the diagnosis of NAFLD is stated by the hepatologist, the patients are referred to the Nutrition Team; they are interviewed by a dietician before the delivery of dietary advice. This gave us the opportunity to test whether the degree of steatosis and the presence of hepatic fibrosis could be related to their spontaneous energy intake and to the proportion of energy from carbohydrate, as reflected by the food quotient.

## 2. Materials and Methods

The dietary interviews were performed by dieticians, who performed a seven-day recall of dietary intakes, using a BillNutIV software for the analysis of the nutrient intakes, in 24 patients with NAFLD, 3 ± 2 months after their hepatic biopsy was performed, and before the deliverance of a dietary advice. Other causes of liver diseases (virus and drugs) were excluded, as were the subjects who declared an excessive alcohol consumption (>20 g/d for women and 30 g/d for men) and the subjects who had known diabetes that might have led them to restrain their carbohydrate intake.

The food quotient, indicator of the proportion of calories from carbohydrates, was calculated as (1.00×% calories from carbohydrates/100) + (0.70×% calories from lipids/100) + (0.81×% calories from proteins/100) [11].

Steatosis was evaluated by a senior pathologist as a percent of hepatocytes containing fat droplets, either macrovacuolar or microvascular, on liver sections stained with HES (hematin-eosin-saffron) of transparietal liver biopsies. The presence or absence of fibrosis (any stage, including cirrhosis) was evaluated histologically by the same pathologist, using Masson's trichromic stain.

The associations between diet variables and steatosis% on the hepatic biopsies were tested by regression analysis, and diet variables were compared according to the presence of fibrosis on the biopsies.

## 3. Results and Discussion

Twenty-four subjects (15 men, 9 women, age 45 ± 13 yrs) were referred to the Nutrition Team after the diagnosis of NAFLD was stated on a liver biopsy. They were overweight (BMI: 29.7 ± 3.8; waist circumference: men 100 ± 7 cm; women 100 ± 8 cm), with abnormal liver tests (ALAT: 72 ± 53 IU/L, GGT: 117 ± 98 IU/L). According to the exclusion criteria, they were not known as diabetic (fasting glycemia: 5.4 ± 0.8 mM), and their alcohol intake was below the recommended limits in France: mean 2 ± 5 g/day.

**Figure 1:**
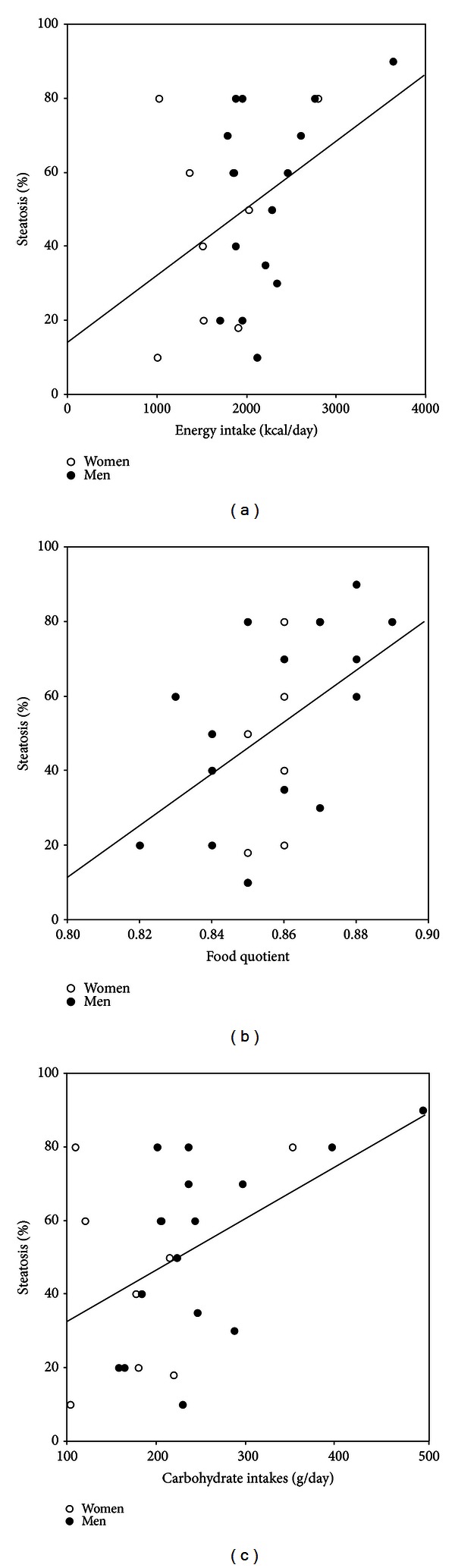
Steatosis% (*y*-axis) as a function of the energy intakes (*x*-axis, (a)), food quotients (*x*-axis, (b)), and carbohydrate intakes (*x*-axis, (c)) in 24 patients with NAFLD (women—open circles, men—closed circles).

The steatosis% was 50.5 ± 25.5 [10–90]. The relations between the steatosis% on the hepatic biopsy and the dietary calorie intake (kcal/day) and the food quotient were both significant (*r* = 0.41 and 0.42, resp., both *P* < 0.05) as depicted in Figures 1(a) and 1(b) (*r* = 0.51, *P* = 0.039 with both by multivariate regression analysis). This confirms that less calories *and less carbohydrates *in the diet are associated with less fat in the liver of the patients with NAFLD. The steatosis% was also correlated to the carbohydrate intakes expressed as grams/day (*r* = 0.49, *P* = 0.015, Figure 1(c)), whereas the correlations with the intakes of lipids (*r* = 0.15), proteins (*r* = 0.30) and simple sugars (*r* = 0.10) were all far from significance. De novo lipogenesis is considered as a small contributor to the accumulation of fat in the whole body [12], but several investigators have reported that it may be an important contributor for liver fat [13–15], and two recent reports using proton magnetic resonance spectroscopy emphasized this importance. Browning et al. demonstrated a greater reduction of hepatic triglycerides after 2 weeks on a low-carbohydrate diet (26 g carbohydrates/day) than during a 50% carbohydrate low-calorie diet in 18 patients with NAFLD [16]. On the other hand, Sevastianova et al. have shown that only three weeks of carbohydrates overfeeding could increase liver fat by +27% contrasted with a +2% body weight gain [17]. In our patients, the relation between liver fat and dietary carbohydrates applied to a more usual range of carbohydrate proportion: our extreme food quotients were 0.82 (33% carb, 44% lipids, 23% protein, and 20% liver steatosis) and 0.89 (58% carb, 30% lipids, 12% protein, and 80% liver steatosis), which suggests that even moderate reductions of dietary carbohydrate may help to reduce liver fat in NAFLD. However, hepatic steatosis is, by itself, a benign condition [18], and the real offends are hepatic inflammation and fibrosis that may not rely on the same mechanisms.

Seventeen of the 24 patients had liver fibrosis, their characteristics are compared to those of nonfibrotic patients in Table 1. In our patients, fibrosis was not related to high calorie intakes, that were even lower (1860 ± 471 kcal/day versus 2380 ± 690 if no fibrosis, *P* < 0.05), or to food quotients, that did not differ (fibrosis: 0.85 ± 0.02, no fibrosis: 0.86 ± 0.02, NS). The older age of the patients with fibrosis may have contributed to both their lower calorie intakes and to their hepatic fibrosis as their exposure to steatosis had a presumably longer duration. These negative results do not question the interest of a well-balanced weight-losing lifestyle intervention, which was proven to improve steatosis and inflammation by a randomized controlled trial [19]. Because our effective was limited, we cannot rule out that very high intakes [20] or some specific forms of carbohydrates like fructose [21] may favor hepatic fibrosis. But by contrasting this with the relation to the steatosis%, we failed to detect a change in the lipid/glucide proportion in our patients with fibrosis; their absolute carbohydrate intakes were even significantly lower as shown in Table 1. On the long term, very low-carbohydrate diets are limited by high attrition rates [22] and a questionable risk of coronary heart disease [23]. We therefore feel that reducing carbohydrate is an interesting track in the field of NAFLD, but it needs caution for a generalized long-term application.

## 4. Conclusions

Hepatic steatosis was related to the energy and carbohydrate intakes in our patients. The role of dietary carbohydrates was detectable in the range of usual intake: 32% to 58% calories from carbohydrates, but the energy intakes and the food quotients were not higher in the patients with hepatic fibrosis, the real offender.More information seems required before considering that reducing carbohydrates from the diet is beneficial on the long term in patients with NAFLD.

## Figures and Tables

**Table 1:** Comparison of the subjects with versus without liver fibrosis.

	With fibrosis	No fibrosis	*P*
*n*	17	7	
Men	10	5	NS
Age (yrs)	49 ± 11	34 ± 12	<0.05
ALAT (IU/L)	50 ± 23	127 ± 69	<0.001
GGT (IU/L)	125 ± 98	95 ± 102	NS
Fasting glycaemia (mmol/L)	5.5 ± 0.7	5.1 ± 0.4	NS
Steatosis% on the hepatic biopsy	45 ± 25	63 ± 21	NS
Body Mass Index (kg/m^2^)	30.6 ± 3.4	27.4 ± 4.0	NS (0.06)
Maximal BMI during life	33.2 ± 3.4	28.7 ± 4.0	<0.01
Waist circumference (cm)	101 ± 8	96 ± 6	NS
Energy intake (kcal/day)	1863 ± 503	2382 ± 73	<0.05
Carbohydrate intake (grams/day)	204 ± 63	290 ± 117	<0.05
Simple sugars (grams/day)	70 ± 40	103 ± 60	NS
Lipid intakes (grams/day)	173 ± 55	194 ± 35	NS
Protein intakes (grams/day)	88 ± 20	110 ± 32	NS (0.06)
Food quotient	0.85 ± 0.02	0.86 ± 0.01	NS
